# The role of B-cells in immunity against adult *Strongyloides venezuelensis*

**DOI:** 10.1186/1756-3305-6-148

**Published:** 2013-05-24

**Authors:** Mohamed A EL-Malky, Haruhiko Maruyama, Saeed A Al-Harthi, Samar N El-Beshbishi, Nobu Ohta

**Affiliations:** 1 Department of Medical Parasitology, Faculty of Medicine, Umm AL-Qura University, Makkah, Kingdom of Saudia Arabia; 2 Department of Medical Parasitology, Faculty of Medicine, Mansoura University, Mansoura, Egypt; 3 Division of Parasitology, Department of Infectious Diseases, Faculty of Medicine, University of Miyazaki, Miyazaki, Japan; 4 Section of Environmental Parasitology, Graduate School of Medical and Dental Sciences, Tokyo Medical and Dental University, Tokyo, Japan

**Keywords:** *Strongyloides venezuelensis*, B cells, JHD knockout, Immunity

## Abstract

**Background:**

*Strongyloides venezuelensis* has been used as a tool and model for strongyloidiasis research. Elimination of *S. venezuelensis* adult worms from mice has been particularly associated with proliferation and activation of intestinal mast cells and eosinophils. To date, the role of B-cells in the protective mechanism against adult *Strongyloides* infection in experimental animals has not been reported in the literature. Therefore, the present study was carried to investigate the role of B-lymphocytes in immunity against adult *S. venezuelensis* infection using mice with a targeted deletion of the JH locus.

**Methods:**

JHD knockout mice with its wild-type Balb/c mice were infected by intra-duodenal implantation of adult *S. venezuelensis*. Fecal egg count, intestinal worm recovery, mucosal mast cells and eosinophils were counted.

**Results:**

At day 11 post infection, parasites in wild-type mice stopped egg laying, while in JHD knockout mice parasites continued to excrete eggs until the end of the observation period, day 107. The higher number of parasite eggs expelled in the feces of JHD knockout infected mice was a consequence of higher worm burdens, which established in the small intestine of these animals. On the other hand worm fecundity was comparable in both groups of mice. Both B-cell-deficient mice and wild-type mice, showed an influx of mucosal mast cells and eosinophils. The absolute numbers in JHD knockout mice were lower than those seen in wild-type mice at day 11, but not to a level of significance. JHD knockout mice could not recover from infection despite the recruitment of both types of cells.

**Conclusion:**

Our findings highlight a role of B cells in mucosal immunity against invasion of adult *S. venezuelensis* and in its expulsion. Therefore, we conclude that B-cells together with mucosal mast cells and eosinophils, contribute to immunity against adult *S. venezuelensis* by mechanism(s) to be investigated.

## Background

Nematode species that colonize the gastrointestinal tract represent a public health problem particularly in tropical and subtropical countries, and are responsible for millions of clinical cases and contributing to many deaths per year [1,2]. Among those, *Strongyloides* infection afflicts 30–100 million people in 70 different countries [3,4]. Accelerated auto-infection, mainly after an alteration in immune status, can cause a syndrome of severe hyperinfection or potentially fatal disseminated strongyloidiasis [5].

Despite the high prevalence and chronic morbidity produced by intestinal nematodes, immunoprotective mechanisms involved in the response against these parasites are not completely understood. Expulsion of parasites from host intestine is the most dramatic form of immunity in intestinal nematode infections [6]. Although the specific effector mechanisms involved in the control of primary nematode infection are not totally understood, type-2 immune responses, through the synthesis of IL-4, IL-5, IL-9 and IL-13, and consequent production of IgE, eosinophilia and mast cells, have been associated with host protection in many experimental models [7,8]. In the specific case of *Strongyloides* infection, the association of type-2 immune response and protection of the host have been reported in human infection and in experimental models. *S. venezuelensis* has been used as a tool and model for strongyloidiasis research [9,10]. Elimination of *S. venezuelensis* adult worms from mice has been particularly associated with proliferation and activation of intestinal mast cells and eosinophils [11-15].

Several studies have demonstrated > 90% reduction in worm count and fecundity of worms in rats [16] and mice [10,17,18] that were infected and challenged with live-larvae of *S. venezuelensis* or *S. ratti* compared to only primary infected animals. It has been suggested that eosinophils, neutrophils and parasite-reactive antibodies were associated with destruction of *Strongyloides* larvae [19-22].

The role of B-cells in primary and challenge infections of larval *S. stercoralis* in mice had been studied [21] and the authors concluded that B-cells are not required in the primary response, yet they are required in the secondary immune response.

To date, a detailed investigation of the role of B-cells in the protective mechanism against adult *Strongyloides* infection in experimental animals has not been reported in the literature. The use of the immunodeficient animals helps to understand the checkpoints in host immunity. Therefore, the present study was carried to investigate the role of B-lymphocytes in immunity against adult *S. venezuelensis* infection using mice with a targeted deletion of the JH locus. This phenotype results in the absence of B-cells and subsequently antibody production [23].

## Methods

### Parasites and animals

Male Balb/c mice and Wistar rats were purchased from Kyudo (Kumamoto, Japan). JHD knockout mice on a Balb/c background [23] have been purchased from Taconic (Hudson, NY, US).

*S. venezuelensis* has been maintained in male Wistar rats in the Division of Parasitology, Department of Infectious Diseases, University of Miyazaki, Japan [24]. Mice were infected by surgical implantation of adult *S. venezuelensis* worms in the small intestine. For adult worm implantation, the upper half of the small intestine of Wistar rats, 8–10 days post-infection was opened longitudinally and washed with phosphate-buffered saline (PBS), followed by incubation in PBS at 37°C for 80 min. Adult worms that emerged from the intestine were washed with sterile PBS and adjusted to the appropriate number. Adult worms suspended in 500 μl of PBS were inoculated into the duodenum of the ether-anesthetized mice (1500/mouse) [25]. All experimental animals were kept and handled under the guidelines of the Animal Experiment Committee, University of Miyazaki, Japan.

### Fecal egg count

Feces were collected daily, starting 2 days after surgical implantation of *S. venezuelensis* worms. Individual feces were weighed separately, and suspended in water. Eggs in small portions of each sample were counted under a microscope, and the number of eggs per gram of feces (EPG) was determined for each sample [9].

### Recovery of adult worms from the intestine

Worms were recovered at day 5 and 11 from the small intestine of each infected mouse according to the method described before [14]. Briefly, the upper half of the small intestine from each infected mouse was removed after sacrifice, washed, cut open longitudinally, and incubated in PBS at 37°C for 4 h. Worms that emerged from the intestinal tissue were quantified by stereomicroscopy.

### Histology

Mucosal mast cells and eosinophils were counted at the time of worm expulsion in wild type mice. For JHD knockout mice, a group of 5 mice were sacrificed for histological examination at day 11 (same day of wild type scarification), and the rest were sacrificed at the end of the observation period (day 107).

For mucosal mast cells, tissues of the small intestines were fixed with Carnoy’s fixative, and paraffin-embedded sections were stained with Alcian blue, pH 0.3, and Safranin-O, pH 0.1 [26]. The number of intra-epithelial mast cells were counted in 50 villus-crypt units (VCU) and expressed as mast cell numbers per 10 VCU.

For eosinophils, tissues were fixed in acetone, and paraffin-embedded sections were stained with hematoxylin followed by 1% water soluble Biebrich Scarlet (Sigma) for 5 min [27]. The number of eosinophils in the small intestine was counted in 50 VCU and expressed as eosinophil number per 10 VCU.

### Statistical analysis

Experiments consisted of five mice per group and all experiments described were performed at least twice. SPSS software was used for data analysis. Descriptive statistics including the mean ± standard deviation (SD), and median values were used. A non-parametric Mann–Whitney test was used to test for significant differences between groups. The data were considered significant if *P* values were less than 0.05.

## Results

**Figure 1:**
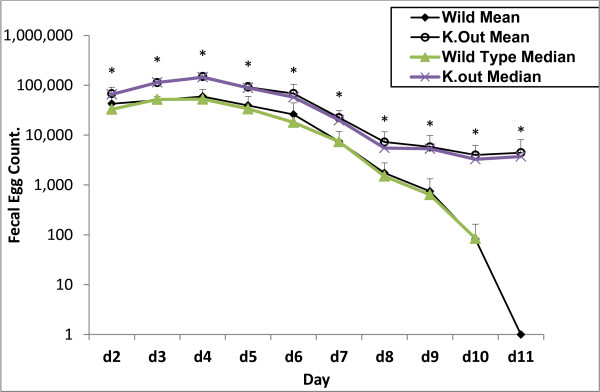
**Fecal egg count after surgical implantation of *S. venezuelensis *adult worms in wild-type and JHD knockout mice.** Mice were infected with 1500 adult worms, and EPG were counted from 2nd day after implantation. All values are mean ± SD, and median values were also included. ^*^P < 0.05.

In this study, we surgically implanted adult worms to examine the mucosal protection against adult *S. venezuelensis*. After the same number of adult worms (1500/mouse) was implanted in the small intestine, both groups of mice started to lay eggs from the 2nd day. The fecal egg count was significantly higher in JHD knockout mice compared to wild-type mice. Moreover, parasites from wild-type mice stopped laying eggs by day 11 after implantation, while parasites from JHD knockout mice continued to lay eggs till the end of the observation period, day 107 (Figure 1).

The kinetics of *S. venezuelensis* infection in mice revealed that there was a statistical difference in the numbers of worms recovered from the small intestines of knockout mice at day 5 after surgical implantation compared to wild type mice (Table 1). Worm fecundity was comparable in both groups of mice (Table 1). Day 11 post infection no worms were recovered from wild type mice.

**Table 1:** **The number of worm recovery, fecal egg count and fecundity 5 days after surgical implantation of *S. venezuelensis *adult worms in wild-type and JHD knockout mice**

	**Wild type mice**	**JHD knockout mice**
Worms recovered	325 ± 45	749 ± 50^*^
	(317)	(747)
FEC	39383 ± 20397	91896 ± 23824^*^
	(33645)	(88398)
Eggs/worm/gm feces	117 ± 47	121 ± 24
	(106)	(118)

All values are mean ± SD.

Numbers given between parentheses indicate the median value.

^*^P < 0.05.

*FEC* fecal egg count.

At day 11, all wild type mice and one group of JHD knockout mice were sacrificed for histological examination. The number of mucosal mast cells (MMC) and eosinophils were comparable in both types of mice (Table 2).

**Table 2:** **The number of mucosal mast cells and eosinophils 11 days after surgical implantation of *S. venezuelensis *adult worms in wild-type and JHD knockout mice**

**Cell count**	**Wild-type mice**	**JHD knockout mice**
**MMC/10 VCU**	142 ± 21	123 ± 15
	(151)	(120)
**Eosinophils/10 VCU**	38 ± 4	34 ± 3
	(38)	(33)

All values are mean ± SD.

Numbers given between parentheses indicate the median value.

*MMC* mucosal mast cells.

*VCU* villus-crypt units.

By the end of the observation period (day 107), MMC and eosinophils in JHD knockout mice were still present, with insignificantly lower counts compared to day 11 counts of the same group (Table 3).

**Table 3:** **The number of mucosal mast cells and eosinophils in JHD knockout mice 11 and 107 days after surgical implantation of *S. venezuelensis *adult worms**

**Cell count**	**JHD knockout mice**	**JHD knockout mice**
	**Day 11**	**Day 107**
**MMC/10 VCU**	123 ± 15	0.7 ±0 .4^*^
	(120)	(0.7)
**Eosinophils/10 VCU**	34 ± 3	0.5 ± 0.4^*^
	(33)	(0.6)

All values are mean ± SD.

Numbers given between parentheses indicate the median value.

^*^Significant difference vs. day 11 (*P* < 0.05).

*MMC* mucosal mast cells.

*VCU* villus-crypt units.

## Discussion

Despite the high prevalence and chronic morbidity produced by nematodes, immunoprotective mechanisms involved in the response against these parasites are not completely understood. In this study, we utilized mice with well-characterized mutations that disabled humoral immunity, in order to determine its role in host protection against adult *S. venezuelensis*.

In the absence of functional B-cells, JHD mice excreted significantly higher FEC compared to wild-type mice up to day 11 after infection. Parasites from wild-type mice stopped egg laying by day 11, while parasites from JHD mice continued to excrete eggs till the end of the observation period, day 107. Although FEC is the only parasitological parameter of immunity that can be obtained sequentially and regularly in the same animal over the course of an infection, it does not strictly reflect the fecundity of the female worm population [28] as many other factors may affect the FEC. Determining the number of eggs in utero is a better index of decreased fecundity. Worm fecundity is determined by dividing the total eggs by the total number of adult worms recovered from the small intestine. Since *S. venezuelensis*-infected hosts have only female worms in the small intestine, worm fecundity can be estimated by dividing the number of eggs eliminated in feces by the number of worms recovered from the intestine of each mouse. At day 5 post infection, the kinetics of *S. venezuelensis* revealed that there was statistical difference in the numbers of worms that get established in the small intestines of knockout mice compared to wild type mice. At day 11 post infection, no worms were recovered from wild type mice indicating that all worms established had been expelled. From these data, it is clear that the higher number of parasite eggs excreted in the feces of JHD knockout infected mice was a consequence of higher worm burden in the small intestine of these animals. On the other hand worm fecundity was comparable in both groups of mice.

It is well known that in *S. venezuelensis* infection, mastocytosis and eosinophilia are associated with worm expulsion [11,12,14,15]. Worm expulsion was impaired in mice deficient in the IL-3 gene [11,29]. In these mice, MMC were completely absent and *S. venezuelensis* continued to parasitize the intestine for more than 50 days*.*

In the current study, both B-cell-deficient mice and wild-type mice, showed an influx of MMC and eosinophils. The absolute numbers in JHD knockout mice were lower than those seen in wild-type mice at day 11, but not to the level of significance.

JHD knockout mice could not recover from infection despite recruitment of both types of cells and their persistence until the end of the observation period. Therefore, it is clear that mucosal mastocytosis and eosinophilia are not solely responsible for worm expulsion and other effector mechanisms must be involved in the expulsion process. It is possible that the defect in worm expulsion following surgical implantation of adult *S. venezuelensis* results from the failure of mast cells and eosinophils to degranulate and release their effector mediators. We previously suggested a role of secretory IgA in conjugation with eosinophils in immunity against adult worm invasion and expulsion [14]. Furthermore, the role of IgE in mast cell degranulation remains equivocal; some authors claim IgE plays a role [30], while others deny any role for IgE [31]

To the best of our knowledge, we are the first to report a role for B cells in mucosal immunity against primary invasion of adult *S. venezuelensis* and in its expulsion. It appears that B cells play a critical role in the elimination of adult *S. venezuelensis* by antibodies or other mechanisms that remain to be fully investigated.

## Conclusion

Our findings highlight a role of B cells in mucosal immunity against invasion of adult S. venezuelensis and in its expulsion. Therefore, we conclude that B-cells together with mucosal mast cells and eosinophils, contribute to immunity against adult S. venezuelensis by mechanism(s) to be investigated.

## Competing interests

The authors declare that they have no competing interests.

## Authors’ contributions

ME, HM, SA and NO designed the experiment. ME, SA and SE performed lab work and drafted the manuscript. All authors read and approved the final version of the manuscript.
